# Breast cancer associated CD169^+^ macrophages possess broad immunosuppressive functions but enhance antibody secretion by activated B cells

**DOI:** 10.3389/fimmu.2023.1180209

**Published:** 2023-06-19

**Authors:** Frida Björk Gunnarsdottir, Oscar Briem, Aida Yifter Lindgren, Eva Källberg, Cajsa Andersen, Robert Grenthe, Cassandra Rosenqvist, Camilla Rydberg Millrud, Mika Wallgren, Hannah Viklund, Daniel Bexell, Martin E. Johansson, Ingrid Hedenfalk, Catharina Hagerling, Karin Leandersson

**Affiliations:** ^1^ Cancer Immunology, Department for Translational Medicine, Clinical Research Center, Lund University, Malmö, Sweden; ^2^ Translational Cancer Research, TCR, Medicon Village, Lund University, Lund, Sweden; ^3^ Sahlgrenska Center for Cancer Research, Department of Biomedicine, University of Gothenburg, Gothenburg, Sweden; ^4^ Division of Oncology, Department of Clinical Sciences Lund, Lund University, Lund, Sweden; ^5^ Division of Clinical Genetics, Department of Laboratory Medicine Lund, Lund University, Lund, Sweden

**Keywords:** breast cancer, macrophage, CD169, tolerance, type I IFN, B cell, TLS

## Abstract

CD169^+^ resident macrophages in lymph nodes of breast cancer patients are for unknown reasons associated with a beneficial prognosis. This contrasts CD169^+^ macrophages present in primary breast tumors (CD169^+^ TAMs), that correlate with a worse prognosis. We recently showed that these CD169^+^ TAMs were associated with tertiary lymphoid structures (TLSs) and T_regs_ in breast cancer. Here, we show that CD169^+^ TAMs can be monocyte-derived and express a unique mediator profile characterized by type I IFNs, CXCL10, PGE_2_ and inhibitory co-receptor expression pattern. The CD169^+^ monocyte-derived macrophages (CD169^+^ Mo-M) possessed an immunosuppressive function *in vitro* inhibiting NK, T and B cell proliferation, but enhanced antibody and IL6 secretion in activated B cells. Our findings indicate that CD169^+^ Mo-M in the primary breast tumor microenvironment are linked to both immunosuppression and TLS functions, with implications for future targeted Mo-M therapy.

## 1 Introduction

Macrophages are a heterogeneous population of innate immune cells. They can be divided into resident macrophages, originating from the yolk sac, liver, or bone-marrow during the fetal stage (1, 2), or recruited macrophages that are monocyte-derived (2, 3). The characteristic chronic inflammatory microenvironment in a tumor result in the majority of tumor-associated macrophages (TAMs) being monocyte-derived recruited macrophages (Mo-M) (2–4). TAMs are generally associated with worse prognosis for cancer patients (3). However, tumor infiltration of resident macrophages as an alternative source of TAMs has also been shown (2–4). The tumor microenvironment may affect the polarization of TAMs differently, leading to a plethora of subclasses of TAMs with a range from pro- to anti-inflammatory functions. To successfully target TAMs in cancer patients, it is becoming urgently important to understand the biology of various TAM subpopulations with regard to origin, phenotype, function, and the microenvironmental signals (localization, cellular microenvironment, or tumor type) that affect these traits.

Macrophages are generally associated with a worse prognosis in cancer patients. There is however one clear exception, the lymph node CD169^+^ resident macrophages. Presence of resident CD169^+^ macrophages in lymph nodes has been correlated to an improved prognosis in patients with a variety of cancer types (5–9). The exact role of CD169^+^ macrophages in cancer patients remains unknown. Lymph node resident CD169^+^ macrophages can be divided into two distinct populations, the subcapsular sinus-macrophages (CD169^+^CD163^-^) and the medullary macrophages (CD169^+^CD163^+^), with somewhat varying functions (10, 11). While CD169^+^ subcapsular sinus macrophages are derived from fetal yolk sac or recruited from monocytes during adult life, less is known about the origin of CD169^+^ medullary macrophages (12, 13). The CD169^+^ medullary resident lymph node macrophages are efficient at sensing lipids, pathogen clearance, phagocytosis, and at inducing tissue destruction (13, 14). The role of CD169^+^ subcapsular sinus macrophages is to act as gatekeepers for soluble, lymph-borne, particulate antigens (virus, bacteria or tumor antigens), to deliver antigens to activate B cells present in the lymphoid follicles, and they are also assigned as crucial antigen-presenting cells (APCs) for high-affinity B cell responses (15, 16). In mice, lymph node CD169^+^ macrophages have been associated with both activating (B, T and NK cell activation), and regulating (T_regs_) immune responses (17–21). In viral infections CD169^+^ subcapsular sinus macrophages induce type I IFNs that promote PDL1 expression, resulting in a local T cell exhaustion (20).

We recently showed that CD169^+^ macrophages are found in primary breast tumors (CD169^+^ TAMs), co-localize with the expression of PDL1 (9) and are spatially associated with tertiary lymphoid like structures (TLLS) and T_regs_ (22). While the CD169^+^ TAM/TLLS infiltration in primary tumors associated to a worse prognosis for breast cancer patients, their presence in metastatic lymph nodes were contrastingly associated to a beneficial prognosis (9, 22). These intriguing findings led us to here investigate the role for CD169^+^ TAMs in the primary breast tumor environment and their functional relation to other infiltrating immune cells. We show that human CD169^+^ TAMs in breast cancer can be monocyte-derived macrophages with broad immunosuppressive functions. In conjunction with activated B cells however, they promote B cell antibody and IL6 secretion. Our findings illuminate the role for CD169^+^ TAMs in primary breast cancers and may explain the spatial association between CD169^+^ TAMs and TLSs found in primary tumors and lymph node metastases.

## 2 Material and methods

### 2.1 Breast cancer patients and tumor tissue microarray

Two breast cancer patient cohorts were used for this study, hereafter referred to as the large and small cohort. The large breast cancer cohort presented in this study consisted of 304 patients diagnosed with locally advanced, inoperable, or metastatic breast cancer in Sweden between 2002 and 2007 included in the randomized phase III trial (TEX) (23). A detailed description regarding the trial and the patient cohort has been described previously (22, 24–26). Ethical approval was obtained from corresponding Regional Ethics committees in Sweden of each of the clinics involved in the trial (23–26). Primary tumor material from 231 patients, ages ranging from 27 to 71 years of age, was included in the final analysis due to missing clinicopathological information or low quality of TMA cores for the remaining cases.

The small breast cancer cohort presented in this study consisted of 23 patients diagnosed with invasive primary breast cancer with lymph node and/or distal metastasis, at the South-Swedish Health Care Region between 1976-2005. The clinical material was collected retrospectively from paraffin embedded tissue. Ethical approval was obtained from Regional Ethic committee Lund, Sweden (Dnr 2010/477), according to the Declaration of Helsinki. ER-positivity was defined as >10%, in line with current diagnostic routines in Sweden. Cores from primary tumor, lymph node metastasis and/or distal metastasis were collected and mounted in a tissue microarray (TMA).

### 2.2 Immunohistochemistry

The cores were 1 mm Ø (small cohort) or 0.6 mm Ø (large cohort), and blocks were sectioned at a thickness of 4 µm prior to mounting. TMA sections were automatically pre-treated using the PT Link system and then stained in an Autostainer Plus (DAKO) at pH9 with an overnight staining protocol. Immunohistochemical (IHC) staining was performed on sections using antibodies specific for B-cells (CD20; dilution 1:100; Abcam; clone L-26), T-cells (CD3; dilution 1:100; Abcam; clone 11084), CD169^+^ macrophages (CD169^+^; dilution 1:100; Invitrogen; clone SP216), NK-cells (CD56; dilution 1:100; Novus Biologicals (Centennial, CO, USA); clone NBP2-34280) and a TripleStain IHC kit was used (Abcam, Cambridge, UK). For double CD169/PDL1 staining of xenografts the antibodies anti-CD169 (dilution 1:500, Spring M5160) and anti-PDL1 (dilution 1:500, Cell Signaling 29122) and as secondary antibody staining protocol, a Double Stain Polymer Kit from Nordic Biosite (anti-mouse HRP (brown) and anti-rabbit AP (pink)) was used according to the manufacturer´s guidelines. The glass slides were fixed and mounted using xylene and Cyto Seal (DAKO). All material was scanned using Aperio slide scanner (Leica Biosystems). The material could then be viewed in Aperio ImageScope (v.12.4.3.5008). Separate staining and annotation for CD3 (T cells) had been performed previously (26). For immunofluorescence (IF), anti-mouseCD169 (Alexa488-conjugated; clone 3D6.112; Biolegend) and -F4/80 (Alexa647-conjugated; clone BM8; Biolegend) was used on frozen sections from mouse.

### 2.3 Animal procedures and the NSG co-xenograft model

The paraffin embedded NSG co-xenograft material presented in this study, originated from our previously performed NSG co-xenografts (27). Briefly, female 8-week-old NSG mice (NOD.Cg-Prkdc(scid)Il2rg(tm1Wji)/SzJ strain, The Jackson Laboratory, USA) were housed in a controlled environment. Mice were anesthetized by isoflurane and injected with human breast cancer cells (SUM159) or (MDA-MB-231) at 1x10^6^ cells/mouse on the right flank, alone or in combination with primary human monocytes (1x10^6^ cells/mouse) as previously described (27). Tumors were excised on day 21 after injection and subsequently fixed in 4% paraformaldehyde and embedded in paraffin. Five (N=5) mice were used in each group. All procedures were approved by the regional ethics committee for animal research at Lund University, Sweden (M11-15). Frozen sections of Balb/c spleen and 4T1-tumors were used for the IF, approved by the regional ethics committee for animal research at Lund University, Sweden (approval M149-14). For the 4T1-model, in brief 1x10^5^ 4T1 cells were injected in the mammary fat pad of a Balb/c mouse and dissected on day 21. The animal work was performed in accordance with the ARRIVE reporting guidelines.

### 2.4 Isolation of primary human immune cells

Ethical permit for the use of human leukocytes was obtained from the regional ethical committee at Lund University (Dnr 2021/04792). Concentrated leukocytes were obtained from healthy donors. Ficoll-Paque Plus (GE Healthcare Bio-sciences) gradient was used to isolate peripheral blood mononuclear cells (PBMC). Monocytes, T cells, B cells and NK cells were isolated from PBMCs by magnetic cell sorting (MACS) using: Classical Monocyte Isolation kit, human; anti-CD3-FITC anti-FITC isolation for T cells, Naïve CD4^+^ T cell isolation kit, human; B cell isolation kit II, human; and NK cell isolation kit, human (Miltenyi Biotec), according to manufacturer’s protocol. T_regs_ were isolated using Dynabeads™ Regulatory CD4^+^CD25^+^ T cell kit (Invitrogen Thermo Fisher Scientific).

### 2.5 Cell cultures and Compounds

Monocytes were differentiated into M1-like, M2-like or M2/type I IFN induced CD169^+^ macrophages, in OptiMEM supplemented with penicillin (100 U/ml) and streptomycin (100 μg/ml) using recombinant human (rh) GM-CSF (10 ng/ml) for M1-like macrophages and rhM-CSF (10ng/ml) for M2-like and CD169 expressing macrophages for 5 days, followed by polarization for 2-3 days using: LPS (100ng/ml) and rhIFNγ (20 ng/ml) for M1-like; rhIL-4 (20 ng/ml) for M2-like; and rhIL-4 (20 ng/ml) and IFNα (670 units/ml) for CD169 expressing macrophages. Macrophages were grown in low adherent plates and harvested using non-enzymatic cell dissociation buffer (Sartorius). All cytokines were from R&D Systems, except for IFNα from PBL assay Science, USA. For co-culture experiments, primary macrophages were harvested on day 7 of culture, reseeded in 96 well plates, and incubated with freshly isolated lymphocytes. All co-cultures were performed in OptiMEM media. For T cell suppression assay (TSA); naïve CD4^+^ T cells were activated using CD3/CD28 Dynabeads™ (Gibco), and then plated with macrophages at stimulator-responder ratio ranging from 1:2 to 1:8. For mixed lymphocyte reaction (MLR), macrophages and T cells were plated at a stimulator-responder ration ranging from 1:1 to 1:100, without addition of Dynabeads™. For B cell and B/T cell co-cultures, macrophages and lymphocytes were plated at a stimulator-responder ratio 1:5, without addition of Dynabeads™. Cells were incubated at 37°C for 5 days. For B_reg_ cell differentiation culture, macrophages were cultured with B cells for 2 days, while for B cell activation (plasma cell differentiation cultures), B cells were pretreated with anti-IgM for 4 hours, whereafter macrophages were co-cultured with B cells for 6 days as previously described (28), and as positive control for plasma cell differentiation CpG (2.5 μg/ml) (Invitrogen), IL-21 (50 ng/ml) and CD40L (1 μg/ml) (R&D Systems) was added. Inhibitors for HLA-G (10 μg/ml) (HLA-G monoclonal antibody, Thermo Fisher) and PDL1 (10 μg/ml) (Atezolizumab, Chemtronica AB) were added on first day of incubation and on day 3. ^3^H incorporation was measured using 1 μl Ci [methyl3H] Thymidine (PerkinElmer) for 18h, a MicroBeta Filtermat-96 Cell Harvester (PerkinElmer) and a Wallace 1450 MicroBeta TriLux Liquid Scintillation and Luminescence counter (PerkinElmer).

### 2.6 Functional cell assays

For cytotoxicity assay, lactate dehydrogenase (LDH) activity was measured using a Cytotoxicity detection kit (Roche Diagnostics) according to manufacturer’s protocol. For pinocytosis assay cells were incubated with 0.25 mg/ml FITC-Dextran (Sigma-Aldrich) at 37°C for 20 minutes and subsequently analysed using flow cytometry. TLR3 agonist Polyinosinic-polycytidylic acid sodium salt (Poly(I:C)) (20 μg/ml) (Sigma-Aldrich) was added to Mo-M cultures on day 5. For migration assay of T cells, T_regs_ and B cells, a SPLInsert™ Hanging 3μm pore size (SPL Life Sciences) migration chamber was used. 2x10^5^ isolated T cells, T_regs_ or B cells were allowed to migrate towards conditioned media from M2 or M2/IFN treated CD169^+^ Mo-M for 18h h, or serum as positive control, with subsequent 4% PFA fixation of transmigrated cells and subsequent Cytospin with H/E staining was performed prior to counting. For cell lines, MDA-MB-231 (ATCC) and SUM159 (a kind gift from Professor S. Ethier (27) and bought from BioIVT, NY, US) TNBC breast cancer cells were used.

### 2.7 Flow cytometry, chemokine and cytokine assays

For flow cytometry, FcR Blocking Reagent (Miltenyi Biotec) and antibodies found in **Supplementary Table 2** were used. All antibodies used were purchased from BD Biosciences and samples were run on a FACS Verse flow cytometer (BD Biosciences) with data analysis performed using FlowJo (Tree Star). Supernatants from macrophage cultures were collected on day 7-8, and cytokines were measured using a V-PLEX Human Cytokine 36-Plex (Meso Scale Diagnostics), or IL15 and IgG ELISA (R&D Systems), or for measuring levels of TNFα Human Inflammatory Cytokine bead array (BD Biosciences), all according to manufacturer’s protocols. Gating strategies are shown in **Supplementary Data File 1**.

### 2.8 RNA extraction, cDNA synthesis and reverse transcription qPCR (RT-qPCR)

Total RNA was extracted and purified using total RNA purification kit (Norgen Biotek Corp) and RevertAid RT Reverse Transcription Kit (Thermo Scientific) was used to generate cDNA according to manufacturer’s protocols. qRT–PCR was performed in triplicates using Maxima SYBR Green/Rox (Thermo Scientific) and the Mx3005 P QPCR system (Agilent Technologies), and the relative mRNA expression was normalized to *GAPDH*, *SDHA* and *YWHAZ* housekeeping genes and calculated using the comparative Ct method. List of primer sequences can be found in **Supplementary Table 3**.

### 2.9 Nanostring GeoMX

The proteome analyses were performed on CD169^+^ cells adjacent to lymph node metastases from the small TMA cohort using the Nanostring kits; Solid tumor TME kit, Immune cell profiling/IO drug target/Immune activation status/Immune cell typing - cores, together with a labelled CD169 antibody using the Alexa Fluor™ 647 Antibody labeling kit (Invitrogen Thermo Fischer Scientific), all according to manufacturer’s instructions.

### 2.10 Statistical analysis

Student’s t-test, paired ratio t-test or Analysis of variance (ANOVA) according to figure legends were performed using Graph Pad Prism software. Pearson Chi Square and Linear by Linear association were performed using IBM SPSS Statistics version 26 (SPSS Inc). Correlation between *SIGLEC1* expression, the gene corresponding to CD169 and overall survival, and the correlation between *SIGLEC1* and *CXCL10, IL10, IFNA4* and *IFNB1* in the human breast cancer 1097 TGCA database was performed *via* R2: microarray analysis and visualization platform http://2r.amc.nl. Single cell analyses were performed using the public data set of Human breast tumor single cell RNA Seq data from miPanda (https://mipanda.med.umich.edu/gene/Coexpression) (29).

## 3 Results

### 3.1 Spatial association between CD169^+^ TAMs and lymphocytes in breast cancer

**Figure 1:**
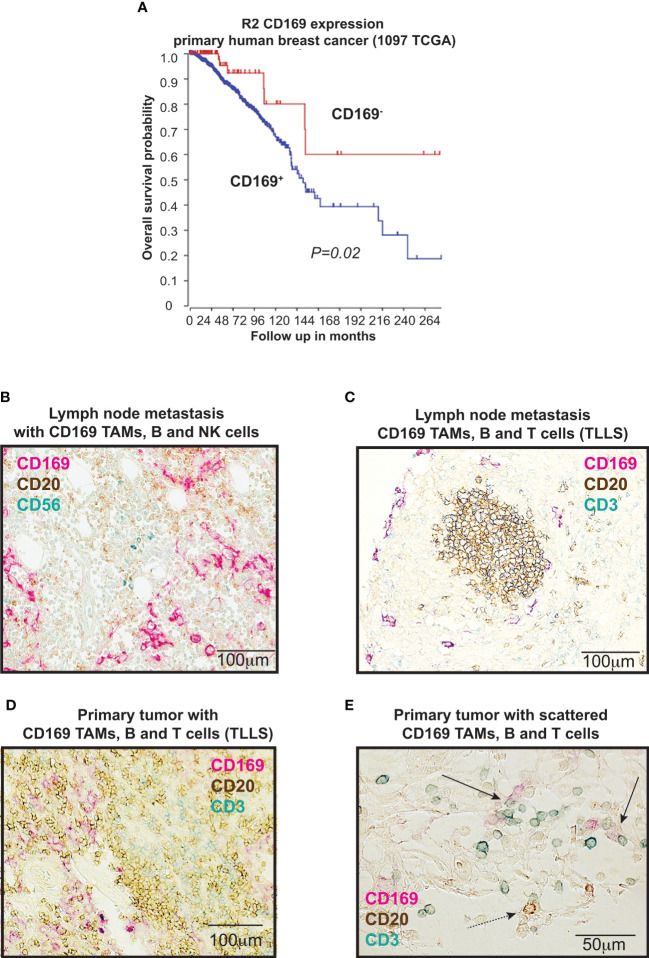
CD169^+^ macrophages and lymphocytes in breast cancers. **(A)** Kaplan-Meier curves illustrating differences in overall survival according to CD169 expression in primary tumors of breast cancer patients using the TCGA data base in R2 (r2.amc.nl). *P* value by log-rank test. **(B-E)** Immunohistochemical (IHC) triple staining of breast tumor tissue array cohort (TMA), using specific antibodies for CD169, CD3 (T cells), CD20 (B cells), and CD56 (NK cells). **(B)** Staining of lymph node metastasis for CD20 (brown), CD169 (red) and CD56 (blue). **(C)** Staining of lymph node metastasis for CD20 (brown), CD169 (red) and CD3 (blue). **(D)** Primary tumor tertiary lymphoid like structure (TLLS) with presence of CD169^+^ tumor associated macrophages (TAMs) stained for CD20 (brown), CD169 (red) and CD3 (blue). **(E)** Magnification of primary tumor with TLLS with presence of CD169^+^ TAMs, T cells and B cells, stained for CD20 (brown), CD169 (red) and CD3 (blue). Solid black arrows point to CD169^+^ TAMs (red) in contact with CD3^+^ T cells (blue), while dashed black arrow points to CD169^+^ TAM in contact with CD20^+^ B cell.

We have recently shown that presence of CD169^+^ TAMs in primary human breast tumors showed evidence for being associated with a worse prognosis (9, 22). These previous results are here supported by data using the TCGA database in R2: Genomics Analysis and Visualization platform (www.hgserver1.amc.nl), where high mRNA expression levels of *SIGLEC1* in primary human breast cancers correlated significantly with worse overall survival (**Figure 1A**) (*P*=0.020). The prognostic impact of *SIGLEC1* using TCGA differed slightly when categorizing into ER^+^ (*P*=0.035) and ER^-^ (*P*=0.098) tumors.

In our recent study we also showed that CD169^+^ TAMs associated with B cells alone, TLLS, T_regs_ and a B_reg_ signature (22). To understand why CD169^+^ TAMs in primary human breast tumors were associated to these cell types and also to a worse prognosis (22), we set out to expand our analysis on the spatial associations between CD169^+^ TAMs and a broader panel of lymphocytes. Representative images for immunohistochemical (IHC) stainings are shown in **Figures 1B–E**. We performed stainings with antibodies specific for: CD169, CD3 (T cells), CD20 (B cells) and CD56 (NK cells) to investigate CD169^+^ TAMs in relation to T cells and B cells (tertiary lymphoid like structures (TLLS; CD20^+^ B cell clusters with CD3^+^ T cells)); or CD169^+^ TAMs in relation to NK cells and B cells. Supporting our recently published data using a large breast cancer cohort (22), CD169^+^ TAMs associated significantly with TLLS also in a small breast cancer tissue array (TMA) test cohort consisting of 23 patients, (**Supplementary Table 4**; Pearson Chi-Square, Linear by Linear association *p=0.048*). CD169^+^ TAMs did however not show any spatial associations with NK cells in the small breast cancer cohort (Pearson Chi-Square, Linear by Linear association *p*=0.449; **Supplementary Table 4**), indicating that NK cells and CD169^+^ macrophages do not usually interact in primary tumors. We therefore did not proceed with further NK cell analysis in the large cohort. However, a significant spatial association between CD169^+^ TAMs in the primary tumors (CD169 PT) and only T cells (CD3) was found using the large breast cancer cohort (**Supplementary Table 5**; Pearson Chi-Square, Linear by Linear association *p=0.018*).

Together with our previous findings we can summarize that tumor infiltrating CD169^+^ TAMs in primary breast tumors are associated with TLLS, T_regs_ and B cells (22) and also with T cells alone as shown here in this study, but not with NK cells.

### 3.2 CD169^+^ TAMs in breast cancer originate from monocytes

**Figure 2:**
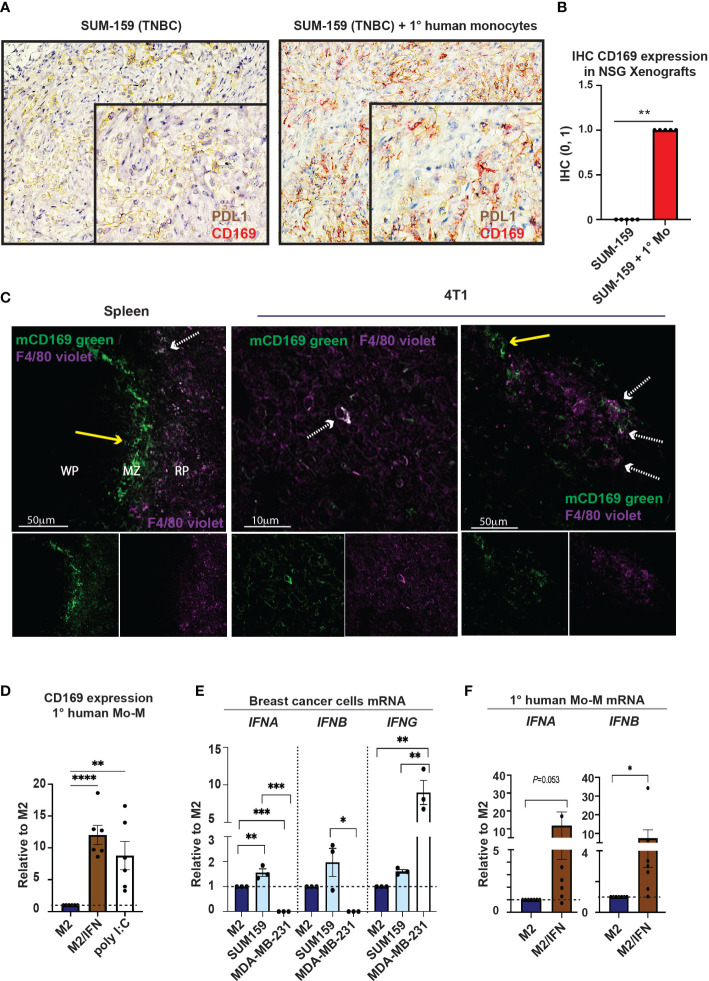
Monocytes can give rise to CD169^+^ TAMs. **(A)** Tumor xenografts in NSG mice were performed previously (27). Primary human monocytes were co-transplanted with SUM159 breast cancer cell lines in NSG mice for 21 days. Controls were transplanted with SUM159 cells alone. CD169^+^ cells (red) were only seen in the SUM159 + monocyte xenografts (right) while PDL1 (brown) was seen in both SUM159 (left) and SUM159 + monocyte (right) xenografts, with CD169/PDL1 co-expression observed in co-transplanted xenografts (right). **(B)** Immunohistochemistry statistics of **(A)** using Mann-Whitney t-test, N=5 in each group, ** p < 0.01. **(C)** Immunofluorescence (IF) staining of a Balb/c mouse spleen (left) and 4T1 tumor model (middle, right). mCD169 shown in green and F4/80 in purple. Arrows point to macrophages only positive for mCD169 (dashed white), or double positive for mCD169 and F4/80 (yellow; approximately 10-20% of the CD169^+^ TAMs), indicating CD169^+^ infiltrating macrophages of both monocyte-derived and possibly resident origin. **(D)** Surface expression of CD169 on primary human monocyte-derived macrophages with addition of type I IFN or the TLR3 ligand Poly(I:C) on day 5 of culture, compared to M2 cultured macrophages as a negative control, N = 6. **(E)** Relative mRNA levels of *IFNB* and *IFNB* in breast cancer cells (SUM159 and MDA-MB-231) and compared to M2 primary human monocyte-derived macrophages (Mo-M) as measured by RT-qPCR, N = 3. **(F)** Relative mRNA levels of *IFNB* and *IFNB* in primary human Mo-M as measured by RT-qPCR, N = 7. For D-E panels: One-way ANOVA multiple comparison Dunnett’s test. For panel F: Ratio paired t-test. Error bars indicate SEM. * p < 0.05, ** p < 0.01, *** p < 0.001, **** p < 0.0001.

We next performed *in vivo* analyses of the cellular origin of CD169^+^ TAMs. To this end, we performed immunohistochemistry on material from our previously published xenograft co-transplantations using primary human monocytes and the human triple negative breast cancer (TNBC) cell lines, SUM159 and MDA-MB-231 (27), in NSG mice (**Figures 2A, B** and **Supplementary Figure 1A**). NSG mice lack functional lymphocytes, have defective macrophages and dendritic cells as a consequence of common gamma chain (γ_c_) deletion, but produce monocytes and neutrophils (30). TNBCs are generally associated with TAM infiltration and PDL1 expression (9, 31, 32). SUM159 and MDA-MB-231 tumor cells in xenografts express PDL1 (**Figure 2A** left and **Supplementary Figure 1A**). When SUM159 tumor cells were co-transplanted with primary human monocytes for 21 days, these monocyte-derived TAMs upregulated CD169 and potentially co-expressed PDL1 (**Figures 2A, B** right), indicating that human CD169^+^ TAMs can be monocyte-derived. The expression of CD169 was however not seen in the other TNBC xenograft using MDA-MB-231 cells (**Supplementary Figure 1A**), indicating that different TNBC tumor cells and microenvironments may have different effects on CD169 upregulation.

To investigate the potential cellular origin of murine CD169^+^ TAMs in breast tumors, we used the immunocompetent and syngeneic murine breast cancer model 4T1 (**Figure 2C** middle and right). In mice, monocyte-derived macrophages (Mo-M) express F4/80 at fluctuating levels during maturation but are often F4/80^-/low^ (33). CD169^+^ resident lymph node subcapsular sinus and spleen marginal zone macrophages respectively, express high levels of mCD169 but lack or express low levels of the murine macrophage marker F4/80, while CD169^+^ resident lymph node medullary/spleen red pulp macrophages express F4/80 (33, 34). We found that approximately 10-20% of CD169^+^ TAMs present were also positive for F4/80 (F4/80^+^; white dashed arrow), but they were mostly negative for F4/80 (F4/80^-^; yellow arrow), indicating infiltrating macrophages of Mo-M origin or possibly resident origin (**Figure 2C** middle and right). Balb/c mouse spleen was used as a staining control (**Figure 2C** left), showing CD169^+^ white pulp (WP) and marginal zone (MZ) macrophages being F4/80^-/low^ (34) (yellow arrow; **Figure 2C** left), and red pulp (RP) macrophages being F4/80^+^ (white dashed arrows; **Figure 2C** left).

In summary, a recruited monocyte-derived origin of human CD169^+^ TAMs in breast tumors is likely, but resident-recruited macrophages should not be disregarded (33). Furthermore, the breast cancer type may affect CD169 upregulation on Mo-M differently depending on the microenvironment and mediators being produced.

### 3.3 Type I IFN is associated with CD169^+^ Mo-M

To understand what causes the unique CD169^+^ phenotype on distinct TAM populations in human breast tumors (9, 22), we next evaluated different inflammatory or tumor-derived mediators on primary human Mo-M, in an *in vitro* M2 tumor microenvironment-setting. We selected relevant mediators that would be able to induce expression of CD169. Type I IFNs have previously been shown to induce CD169 on macrophages (35). CD169^+^ subcapsular sinus macrophages are further themselves high producers of type I IFNs in viral immune responses (20, 36) and found responsible for the PDL1 expression on nearby cells (20), a feature that would fit with the CD169^+^/PDL1^+^ TAM phenotype observed in human breast tumors. As expected, the Mo-M upregulated CD169 specifically in the M2 tumor microenvironment/type I IFN setting (M2/type I IFN) (**Figure 2D**; CD169^+^ Mo-M). The type I IFN inducer TLR3 ligand Polyinosinic:polytidylic acid (Poly(I:C)) also induced CD169 expression on Mo-M (37, 38) (**Figure 2D**). Using qPCR we could show that endogenous *IFNA* and *IFNB* was expressed at very low levels in the breast cancer cell line SUM159, but not in MDA-MB-231 cells (**Figure 2E**), whereas *IFNG* was more expressed in MDA-MB-231 cells (**Figure 2E**). We could further show that CD169^+^ Mo-M are actually capable of expressing type I IFNs (*IFNA* and *IFNB*) themselves *in vitro* whereas M2 macrophages did not (**Figure 2F**), however at lower levels than monocytes and M1 macrophages (**Supplementary Figure 1B**). A possible relationship between type I IFNs and CD169-expression on Mo-M was supported by mRNA data from primary human breast tumors, where mRNA expression for the gene *SIGLEC1* encoding CD169 significantly correlated with *IFNA4* (*P*=6.25e-04) and *IFNB1* (*P*=5.53e-41) (**Supplementary Figure 1C**). Although M1 macrophages expressed type I IFNs, they did not upregulate CD169 (**Figure 2D**, **Supplementary Figure 1B** and **Supplementary Data File 1**), indicating the type I IFN primarily led to CD169 upregulation in an M2/IFN environment like tumors.

Together this indicates that CD169 can be induced on recruited monocyte-derived macrophages (CD169^+^ Mo-M) in a breast tumor microenvironment, and that this is associated with type I IFN production.

### 3.4 CD169^+^ Mo-M have a nique phenotype

**Figure 3:**
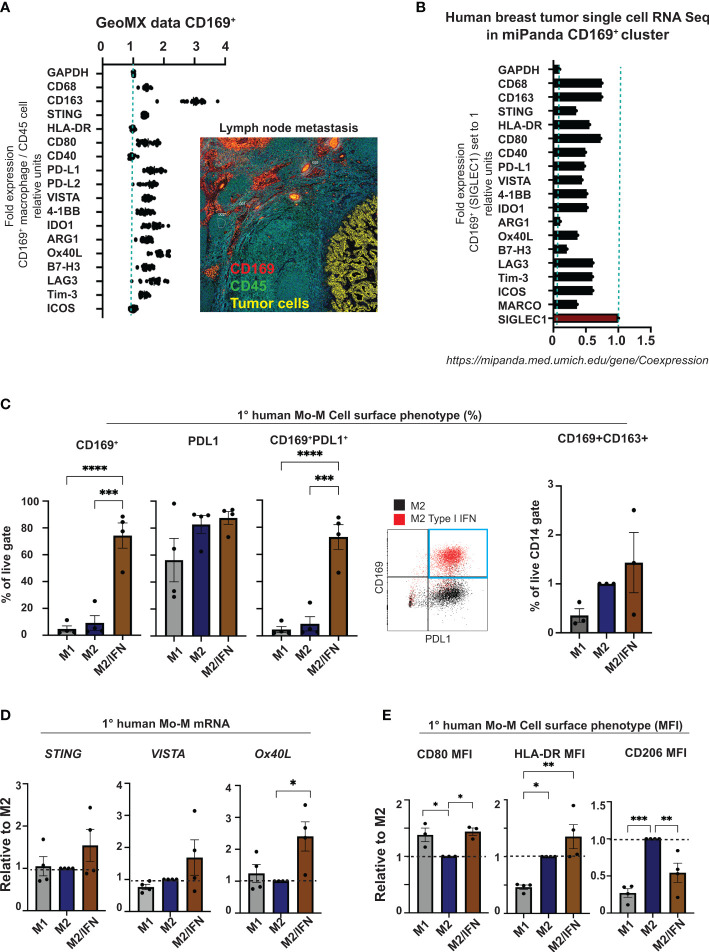
CD169^+^ macrophage phenotype analysis. **(A)** Nanostring GeoMX proteome analysis of CD169^+^ macrophages in lymph node with breast cancer metastases. Regions of CD169^+^ macrophages (red) adjacent to metastasis (yellow) in lymph node were chosen, and expression levels of proteins presented in panel **(A)** were compared in relation to their expression in nearby areas of CD45^+^ cells (green) in follicle structures, representing mostly B cells. **(B)** Single-cell RNA Seq data showing the corresponding genes from panel **(A)** in the CD169^+^ macrophage cluster (CD169^+^ TAMs) from the Human breast cancer data set in miPanda (https://mipanda.med.umich.edu/gene/Coexpression) (29). **(C)** Comparison of CD169 surface expression (left), PDL1, co-expression of CD169 and PDL1 (centre) or CD169 and CD163 (right) on primary human monocyte-derived macrophages. Representative dot-plot showing expression of CD169 and PDL1 on M2 treated control primary human macrophages (black) compared to IFN treated (red) (right), N = 4 and N = 3. **(D)** Relative mRNA expression of *STING, VISTA* and *OX40L* on primary human monocyte-derived macrophages (N=4). **(E)** Ratio of MFI of cell surface markers CD80, HLA-DR and CD206 on human primary macrophages with M2 as control, N = 4. For flow cytometry gating strategies see **Supplementary Data File 1**. For figures C-E: One-way ANOVA multiple comparison Dunnett’s test. Error bars indicate SEM. * p < 0.05, ** p < 0.01, *** p < 0.001, **** p < 0.0001.

We next set out to perform a broad phenotypic analysis of CD169^+^ macrophages in primary human breast tumors compared to those in lymph node metastases. Proteome analysis of CD169^+^ macrophages in lymph nodes with breast tumor metastases using Nanostring GeoMX (**Figure 3A**) were compared to gene expression of the CD169^+^ (*SIGLEC1*) clusters in a public data set of Human breasts tumor single cell RNA Seq data from the Michigan Portal for the Analysis of NGS Data (MiPanda) (29) (**Figure 3B**). The primary breast cancer CD169^+^ TAMs were too few to analyse using the chosen Nanostring GeoMX proteome analysis method. We could however show, that CD169^+^ macrophages in association with lymph node metastasis expressed CD163. They also expressed higher levels of the proteins STING, CD80, VISTA, IDO1 and Ox40L, in relation to CD45^+^ cells in general, and the inhibitory co-receptors PD-L1, B7H3, LAG3 and Tim-3. Of note, in the proteome analysis we compared relative protein expression of CD169^+^ macrophages, with CD45^+^ expressing cells located in follicles in general (**Figure 3A**). The majority of CD45^+^ cells in follicle areas are B cells. This can explain the seemingly low expression levels of *HLADR* and *CD40* on primary breast cancer CD169^+^ TAMs in the proteome analysis (**Figure 3A**) since B cells express high levels of HLADR and CD40 in general. HLADR and CD40 is therefore probably expressed at similar levels on CD169^+^ macrophages and B cells, being antigen presenting cells (APCs) (**Figure 3A**). In the Human primary breast tumor single cell RNA Seq data CD169^+^ (*SIGLEC1*) cluster (**Figure 3B**), the corresponding genes were also expressed, shown using the public data set of Human breast tumor single cell RNA Seq data from miPanda (https://mipanda.med.umich.edu/gene/Coexpression (29)), as was the gene for *MARCO*.

The *in vitro* generated CD169^+^ Mo-M showed a similar cell surface phenotype with prominent PDL1 expression (**Figure 3C** and **Supplementary Figure 1D**), slight CD163 expression (**Figure 3C**), slightly higher levels of STING and VISTA (**Figure 3D**), and significantly higher levels of Ox40L (**Figure 3D**), CD80 and HLA-DR (**Figure 3E**), in relation to M2-like macrophages. Indeed, the CD169^+^ Mo-M also showed a mixed macrophage cell surface phenotype representing both M1- and M2-like macrophages (CD14^hi^HLADR^hi^CD80^hi^CD1a^-^CD206^-^PDL1^+^CD163^-/+^) as seen in **Figures 3C, E** and **Supplementary Figures 1E, F** and **Supplementary Data File 1**.

Together this suggests that CD169^+^ macrophages generated from monocytes in a type I IFN tumor microenvironment *in vitro* (CD169^+^ Mo-M), possess a unique phenotype, much resembling CD169^+^ TAMs in breast tumors and CD169^+^ lymph node macrophages.

### 3.5 CD169^+^ Mo-M have a distinctive mediator profile

**Figure 4:**
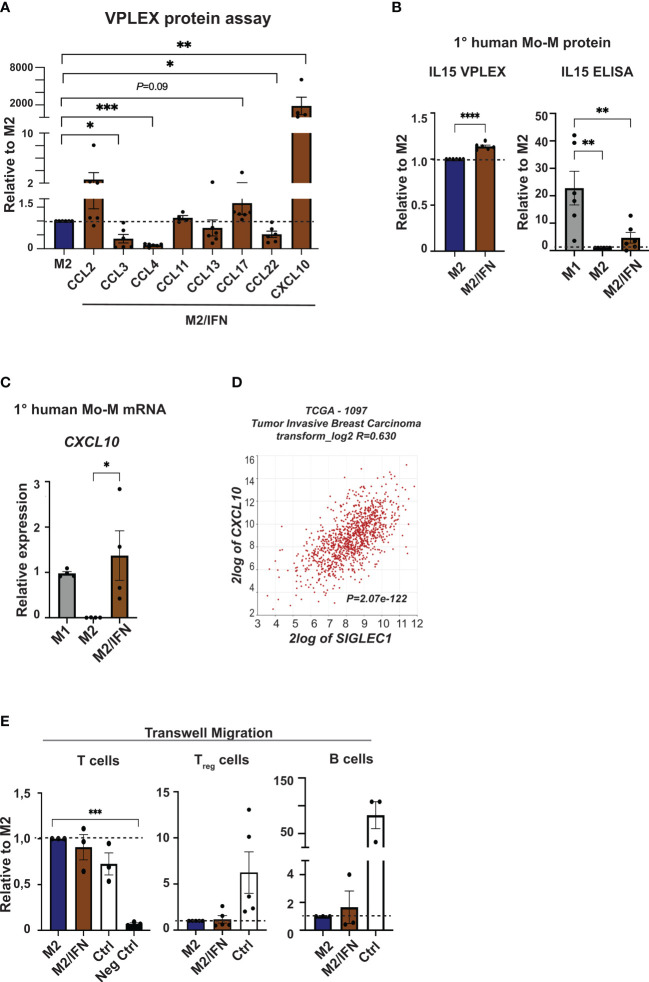
Chemokine profile of CD169^+^ Mo-M. **(A)** Chemokine analyses were performed using V-PLEX, on supernatants from M2 and M2/IFN (CD169^+^ Mo-M). CCL2, CXCL10, CCL11 and CCL17 were induced in the CD169^+^ Mo-M, while CCL3, CCL4, CCL13 and CCL22 were downregulated. **(B)** Cytokine secretion measured with VPLEX of IL15, N = 6. Cytokine secretion of IL15 as measured by ELISA, ratio of concentration with M1 as control, N = 6. **(C)** Relative mRNA expression of and *CXCL10* as measured by RT-qPCR, N = 4. **(D)** *SIGLEC1* association to *CXCL10* mRNA expression in primary tumors of breast cancer patients using the TCGA data base in R2 (r2.amc.nl). **(E)** Transwell migration assay of isolated T cells, T_regs_ and B cells towards serum free conditioned media from M2 or M2/IFN (CD169^+^ Mo-M), with serum as positive control. For panels A-B and E: Ratio paired t-test. For panel D: One-way ANOVA multiple comparison Dunnett’s test. Error bars indicate SEM. * p < 0.05, ** p < 0.01, *** p < 0.001, **** p < 0.0001.

The cytokine and chemokine profile of CD169^+^ MoM was next analysed using the V-PLEX system (**Figures 4A, B** and **Supplementary Table 1**). The *in vitro* type I IFN/M2 tumor microenvironment generated CD169^+^ Mo-M with a distinctive chemokine profile in comparison to paired donor M2 macrophages. Of the 36 cytokines and chemokines analysed, CXCL10 showed a pronounced, significant upregulation and IL15 was slightly upregulated, while CCL2, CCL17 and IL6 were secreted at a notably higher level by CD169^+^ Mo-M although not significant (**Figures 4A, B** and **Supplementary Table 1**). In contrast CCL3, CCL4 and CCL22 were all secreted at lower levels (**Figure 4A** and **Supplementary Table 1**). Using independent methods, we observed that CXCL10 expression was further significantly upregulated in the CD169^+^ Mo-M at mRNA levels (**Figure 4C**) and confirmed results for IL15 and IL6 as measured by ELISA or CBA (**Figure 4B** and **Supplementary Figure 2A**). Interestingly, while M1 macrophages secreted high levels of TNFα, neither M2 nor CD169^+^ Mo-M did (**Supplementary Table 1** and **Supplementary Figure 2B**).

CXCL10 is a chemokine that attracts T cells to tumor sites and is induced by IFNγ and Type I IFNs (39–41). We could show that *CXCL10* is strongly associated with *SIGLEC1* expression in breast cancer specimens in primary tumors using the TCGA database in R2 (r2.amc.nl) (R=0.630, *P*=2.07e-122; **Figure 4D**). Using the Michigan Portal for the Analysis of NGS Data (MiPanda), we saw that *CXCL10* was again highly associated with *SIGLEC1* in primary breast cancer (Pearson correlation *P*=*2.53e-12*) while it was not associated in normal breast tissue (Pearson correlation *P*=*0.66*) (29). Nevertheless, the CD169^+^ Mo-M did neither attract T cells or T_regs_ more than M2-like macrophages *in vitro*, nor B cells according to transwell migration assays performed using supernatant from cultured M2-like macrophages, CD169^+^ Mo-M or a serum control (**Figure 4E**).

In summary, our data indicate that CD169^+^ Mo-M produce high levels of the chemokine CXCL10, but do not attract T cells, T_regs_ nor B cells more than M2-like macrophages, ruling out that the spatial association found between CD169^+^ TAMs, TLLS and T_regs_ is caused by chemotactic processes alone.

### 3.6 CD169^+^ Mo-M have immunosuppressive functions

**Figure 5:**
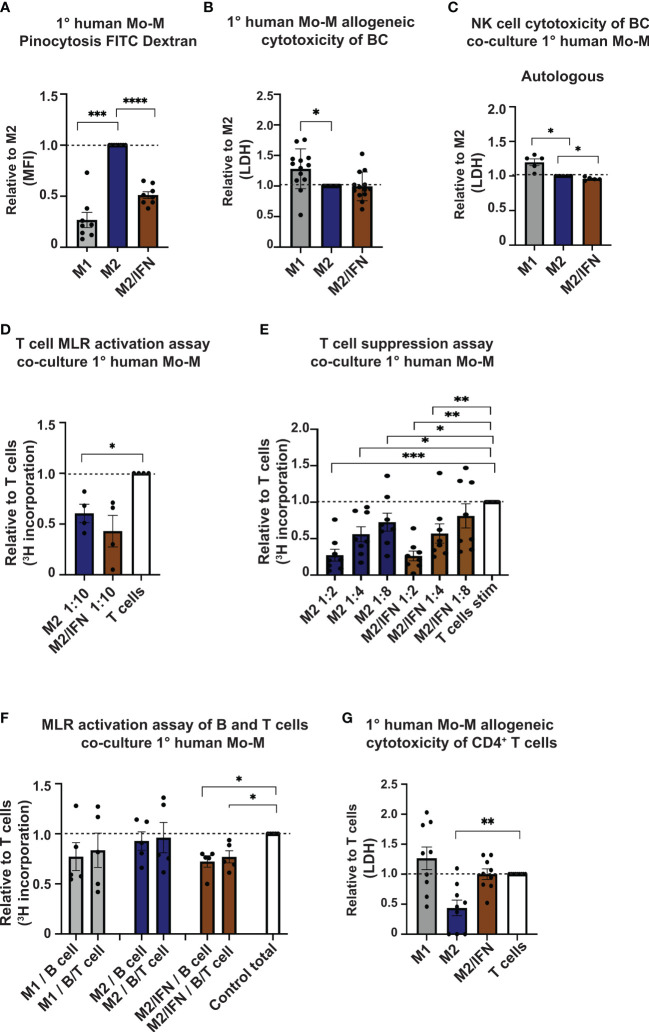
Immune suppressive functions of CD169^+^ Mo-M. **(A)** Pinocytosis capacity as measured by FITC-Dextran uptake with M2 macrophages as control, N = 8. **(B, C)** Allogeneic Cytotoxicity assay as measured by LDH activity released from cytosol of damaged cells. **(B)** Primary human monocyte-derived macrophages cytotoxicity of MDA-MB-231 breast cancer cell line, N = 16. **(C)** Effect of primary human monocyte-derived macrophages on cytotoxicity by primary human autologous NK cells on MDA-MB-231 breast cancer cell line, N=5. **(D)** Allogeneic MLR of primary human monocyte-derived macrophages and primary human CD4^+^ T cells as measured by [^3^H] incorporation at ratio 1:10. Ratio with base T cell [^3^H] incorporation, represented by dashed line, N = 3. **(E)** Allogeneic T cell suppression assay of primary human monocyte-derived macrophages and primary human CD4^+^ T cells activated with CD3/CD28 beads. Dashed line represents base activated T cell [^3^H] incorporation, N = 8. **(F)** Allogeneic MLR of primary human monocyte-derived macrophages and primary human B cells or B cells/CD4^+^ T cells as measured by [^3^H] incorporation at ratio 1:5, N=5. Ratio with base T cell or B cell [^3^H] incorporation, represented by dashed line. **(G)** Cytotoxicity assay as measured by LDH activity released from cytosol of damaged cells of allogeneic primary human monocyte-derived macrophages cytotoxicity on CD4^+^ T cells. N=9. For panels A-F: Ratio paired t-test. Panel G: One-way ANOVA multiple comparison Dunnett’s test due to values 0. Error bars indicate SEM. * p < 0.05, ** p < 0.01, *** p < 0.001, **** p < 0.0001.

We next asked which functional phenotype the CD169^+^ Mo-M generated *in vitro* in an M2/type I IFN tumor microenvironment setting would have. We analysed pinocytic and immune-activation or suppression capacity in relation to T cells, B cells and NK cells. Firstly, the CD169^+^ Mo-M had a significantly reduced pinocytic capacity compared to M2-like macrophages, but still slightly better than M1-like macrophages (**Figure 5A**). Co-culture of macrophages with MDA-MB-231 breast cancer cells, did not show cytotoxic activity for CD169^+^ Mo-M as compared to M1-like macrophages (**Figure 5B**), indicating an M2-like function. Using co-cultures of macrophages and autologous NK cells together with MDA-MB-231 breast cancer cells revealed that presence of CD169^+^ Mo-M significantly reduced NK cell tumoricidal capacity, in contrast to presence of M1-macrophages (**Figure 5C**). The CD169^+^ Mo-M further acted immunosuppressive in relation to T cells (**Figures 5D, E**), a typical M2-like function. Importantly, the CD169^+^ Mo-M even acted immunosuppressive towards non-activated B cells and B/T cell co-cultures, a trait that neither M1- or M2-like macrophages had (**Figure 5F**). This inhibitory effect was not caused by graft versus host cytotoxicity as CD169^+^ Mo-M did not kill allogeneic CD4^+^ T cells (**Figure 5G**).

In summary, given that the CD169^+^ TAMs in breast cancer associate with lymphocytes and TLSs in primary tumors, our functional data propose that CD169^+^ TAMs do not promote NK, T or B cell proliferation or activation, but rather inhibit them.

### 3.7 CD169^+^ Mo-M express immunosuppressive mediators

**Figure 6:**
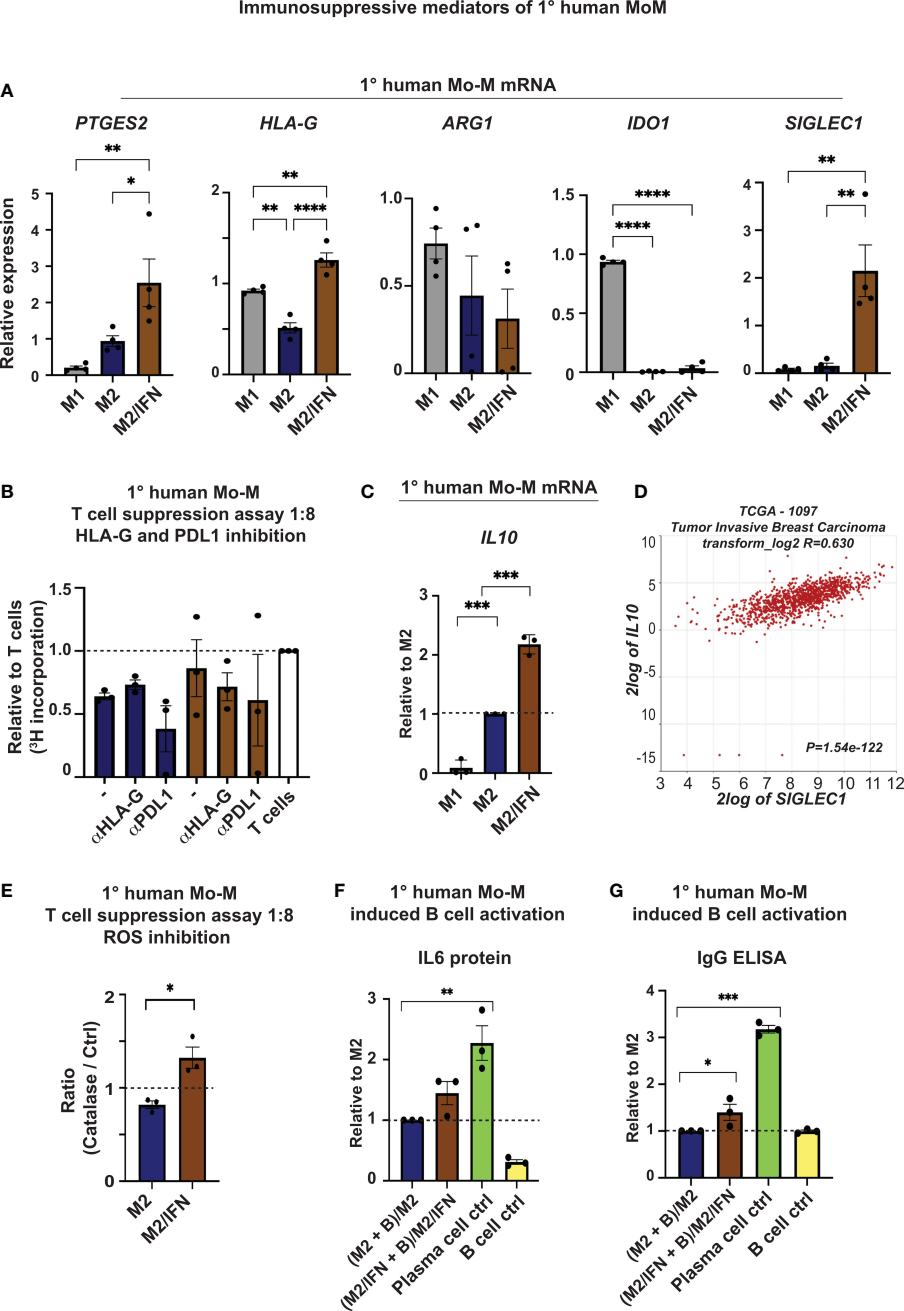
Immunosuppressive mediators of CD169^+^ Mo-M. **(A)** Relative mRNA expression of PGE2 (*PTGES2*), HLA-G (*HLA-G*), Arginase (*ARG1*), Indoleamine 2,3-Dioxygenase 1 (*IDO1*) and CD169 (*SIGLEC1*) as control. N = 4. One-way ANOVA. **(B)** Allogeneic T cell suppression assay of primary human monocyte-derived macrophages and primary human CD4^+^ T cells as measured by [^3^H] incorporation at ratio 1:8 with M2 macrophages (blue) and M2/IFN macrophages (brown), with inhibitors for HLA-G (10 μg/ml) and PDL1 (Atezolizumab, 10 μg/ml). Ratio with base activated T cell [^3^H] incorporation, represented by dashed line, N = 3, Ratio paired t-test. **(C)** Relative mRNA expression of *IL10* as measured by RT-qPCR, N = 3. **(D)** *SIGLEC1* association to *IL10* mRNA expression in primary tumors of breast cancer patients using the TCGA data base in R2 (r2.amc.nl). **(E)** Allogeneic T cell suppression assay of primary human monocyte-derived macrophages and primary human CD4^+^ T cells activated with CD3/CD28 beads as measured by [^3^H] incorporation at stimulator-responder cell ratio 1:8 with and without the ROS inhibitor Catalase. Dashed line represents base activated T cell [^3^H] incorporation without the ROS inhibitor Catalase for each group, N=3. Paired t-test. **(F, G)** B cell activation cultures of primary human monocyte-derived macrophages and anti-IgM stimulated primary human B cells as measured by **(F)** IL6 and **(G)** IgG secretion using ELISA, N=3. One-way ANOVA multiple comparison Dunnett’s test. For all figures: Error bars indicate SEM. * p < 0.05, ** p < 0.01, *** p < 0.001, **** p < 0.0001.

To investigate possible immunosuppressive mediators expressed by the CD169^+^ Mo-M, other than PDL1 and Ox40L (**Figure 3**), we next performed ELISA and qPCR analyses of various NK, T and B cell inhibitory effector molecules (**Figure 6**). The NK and T cell inhibitory mediators PGE2 (*PTGES2)* (42, 43) and HLA-G (*HLA-G)* (44) were both specifically upregulated at mRNA level in the CD169^+^ Mo-M, compared to both M1- and M2-like macrophages (**Figure 6A**). Arginase (*ARG1*) and Indoleamine 2,3-dioxygenase (*IDO1*) were not significantly upregulated at mRNA level in the CD169^+^ Mo-M (**Figure 6A**), also in line with the single cell data in **Figure 3**. The increased level of IDO1 in M1 macrophages can be explained by the fact that IFNγ upregulates IDO1 on M1-like macrophages (45). Inhibition of HLA-G or PDL1 did however not alleviate the suppressive effect that CD169^+^ Mo-M had on T cells (**Figure 6B**), nor affect NK cell cytotoxicity in co-cultures with macrophages and breast cancer cells (**Supplementary Figure 2D**). Instead, the immunosuppressive cytokine *IL10* mRNA was significantly upregulated by CD169^+^ Mo-M as compared to both M1 and M2-macrophages (**Figure 6C**). This finding was supported by a strong correlation between *IL10* and *SIGLEC1* expression in primary tumors from breast cancer patients using the TCGA data base in R2 (r2.amc.nl) (R=0.63; P=1.54e-122) (**Figure 6D**), but not by the IL10 V-PLEX protein analysis data due to low detection levels (N=3; **Supplementary Table 1**), a finding that also could be explained by natural polymorphism in the human *IL10* gene promoter (46). The ROS inhibitor Catalase alleviated the suppressive effect caused by the CD169^+^ Mo-M on T cell proliferation, as compared to M2-like macrophages (**Figure 6E**).

Together this indicates that CD169^+^ Mo-M generated in a tumor microenvironment *in vitro*, act immunosuppressive in relation to NK and T cells *via* typical M2-like mediators (PGE2, ROS and IL10).

### 3.8 CD169^+^ Mo-M promote IgG and IL6 secretion by activated B cells

M2-like mediators (PGE2, ROS and IL10) may affect the accumulation or differentiation of T_regs_. However, *in vitro* CD169^+^ Mo-M did not promote T_reg_ differentiation (**Supplementary Figure 2D**) nor IL10-producing B_reg_ cells (**Supplementary Figure 2E**). We finally co-cultured the CD169^+^ Mo-M with previously activated primary human peripheral blood B cells for six days, to investigate their effect on B cell activation and differentiation. To our surprise, we now found that the CD169^+^ Mo-M promoted antibody (IgG) and IL6 secretion by activated B cells (**Figures 6F, G**), a feature that has previously been associated to spontaneous, local germinal centre formation (47).

This indicates that CD169^+^ Mo-M are immunosuppressive, acting *via* typical M2-like mediators (PGE2, ROS and IL10), but at the same time aid in antibody and IL6 secretion from activated B cells. This would explain the functional reason for being localized near TLSs in breast tumors. It could also indicate that CD169^+^ Mo-M and CD169^+^ TAMs studied here, are indeed functionally similar to CD169^+^ subcapsular sinus macrophages in lymph nodes.

## 4 Discussion

The importance of lymph node resident CD169^+^ macrophages as beneficial immune cells in cancer patients has come to light lately (48). Although their role during viral infections is becoming clearer, there is still a large gap of knowledge regarding their mechanisms of action in cancer patients. Indeed, CD169^+^ lymph node macrophages have been shown to have both immunogenic and tolerogenic functions (15–21, 35, 36, 49), thus more data is needed to understand their involvement in cancer. When CD169^+^ macrophages are present in primary breast tumors (CD169^+^ TAMs), they are closely linked to a worse prognosis (9, 22). It is still unknown whether resident CD169^+^ lymph node macrophages in cancer patients are associated with a beneficial prognosis because of their function, or simply because of their mere presence in lymph nodes at an early tumor stage, thus being a prognostic biomarker. In this study, we aimed to investigate the functional biology of CD169^+^ TAMs in a breast tumor environment, in relation to lymph node resident CD169^+^ macrophages, to understand why CD169^+^ TAMs are associated with a worse outcome.

TAMs in primary tumors are generally associated with recruited Mo-M, of various alternative activation types (2, 3). The majority of TAMs in breast cancer express the typical M2 marker CD163 (50). Only a small minority of TAMs in primary human breast cancers express CD169 (9, 51). Why TAMs would adapt the CD169^+^ phenotype is still unclear. What is clear as shown here however, is that two different TNBC xenografts, presumably having rather similar tumor microenvironments, can give rise to CD169 expression in one situation and not in the other. We suggest that CD169 expression on TAMs can be induced in a certain tumor microenvironment. Type I IFN signalling pathways and signalling leading to type I IFN production may be one cause. TLR3 signalling has previously been shown to induce antitumoral function of macrophages and upregulate secretion of inflammatory cytokines and chemokines such as CXCL10 (38) and type I IFN (37). Tumor specific ligands for TLR3 in the form of damage associated molecular patterns (DAMPs) released from tumor and necrotic cells have also previously been identified (52–54). In this present study we did observe an upregulation of CD169 on M2/Type I IFN treated primary human macrophages, as well as on TLR3 agonist Poly(I:C) treated primary human macrophages. M1 macrophages did however not upregulate CD169 despite showing expression of type I IFNs, indicating that CD169 is upregulated primarily in a tumor M2/IFN microenvironment. Which other specific mediators in the tumor microenvironment that induce CD169 expression on TAMs needs further investigation. We also propose that human CD169^+^ TAMs in breast cancer can be monocyte-derived, just like other TAMs, but a resident origin should not be excluded. This is in line with previous data on hepatocellular carcinoma showing that tumor infiltrating CD169^+^ macrophages originate from monocytes (55), and a study where CD169^+^CD14^+^ TAMs were characterized using CyTOF (56). Another study using the syngeneic 4T1 tumor model, argued that resident CD169^+^ macrophages infiltrate murine mammary tumors (49). We here show using the same model that a proportion of them may indeed rather be monocyte-derived as judged by lack of F4/80, thus indicating a mixed origin. Nevertheless, the expression of CD169 seems to be rare, as compared to other TAM subpopulation markers, and associated with certain breast tumor environments.

The phenotype of CD169^+^ TAMs and CD169^+^ Mo-M indicates similarities to lymph node resident CD169^+^ macrophages. CD169^+^ TAMs may be CD163^+^ just like the proteome and single cell data presented here suggest. However, our experience from working with breast cancers is that infiltrating CD163^+^ TAMs are quite frequent, but CD169^+^ TAMs are not, indicating that only a minority of the CD163^+^ TAMs (M2-like TAMs) are CD169^+^ (9, 51). A recent study presented similar data as ours regarding CD169^+^CD163^+^ TAMs in breast cancer, associating them with a worse prognosis but indicating a connection to TNFα production, which we did not find (57). Instead, we found expression of IL15 and CXCL10. IL15 is important for T and NK cell activity (58) a mechanisms that our CD169^+^ Mo-M did not have *in vitro*, and for antitumor immunity (59) which does not support our prognostic data regarding CD169^+^ TAMs in primary breast cancers (9, 22). As shown here, the CD169^+^ TAMs did not associate with NK cells in breast tumors, thus ruling out an important functional relation between them in breast cancer. Of note, IL15 may also have immunosuppressive effects in a GM-CSF environment (60). CXCL10, which was secreted at high levels in our *in vitro* cultured CD169^+^ Mo-M, has previously been correlated with infiltration of both CD8^+^ and FOXP3^+^ TILs, as well as PDL1^+^ immune cells in breast cancer (61), but also with cell proliferation, migration and epithelial-mesenchymal transition of breast cancer cell lines. Most studies on CXCL10 in breast cancer have focused on CXCL10 expression in the breast cancer cells, rather than the effect of macrophage derived CXCL10. One exception is the tumor driven macrophage expression of CXCL10 in osteolytic bone metastasis that was associated with increased metastases (62). In a study using single cell RNA seq analysis of a breast cancer, *CXCL10* was also found to be expressed in a TAM subpopulation, but these TAMs did not express *SIGLEC1* (51), indicating the possibility of even further macrophage subtypes. Hence, CXCL10 could theoretically explain the correlation between CD169^+^ cells and a worse prognosis found in our patient cohort. We also found slightly elevated levels of CCL17, a chemokine that recently was shown to be expressed in tumor associated tissue resident macrophages of NSCLC and linked to chemoattraction, differentiation and proliferation of T_regs_ (63). A broader analysis of CXCL10 and CCL17 in CD169^+^ TAMs originating from various breast cancer subtypes and stages will be needed to assess their relationship in more detail.

When we analysed the spatial localization of CD169^+^ TAMs in primary tumors in relation to other immune cells, we found that CD169^+^ macrophages were spatially associated with T cells, B cells, tertiary lymphoid like structures (TLLS), immunosuppressive T_regs_, and even a B_reg_ signature (22) in the primary tumor. TLS formation has previously been postulated to be important for anti-tumor immune reactions (64, 65), however we recently published that in patients with advanced breast cancer the opposite is seen (22). We speculated on whether this could be caused by presence of T_regs_ in the TLSs (22), a finding that previously has been described to associate with worse outcome for cancer patients, including breast cancer patients (22, 64, 66, 67). Our *in vitro* data indicate that in contrast to having an anti-tumoral function, the *in vitro* cultured CD169^+^ Mo-M are immunosuppressive in relation to NK cells, T cells and non-activated B cells, with the three most likely inhibitory mediators being PGE_2_, ROS or IL10, probably also involving the inhibitory co-receptors B7H3, LAG3 and Tim-3, here shown to be expressed by the CD169^+^ macrophages. The immunosuppressive mechanism of CD169^+^ TAMs is supported by a study using the CD169-DTA 4T1 tumor model, showing that CD169^+^ macrophages induce tumor progression (49). A similar finding was shown in lung cancer models (68), but the opposite was found in glioblastoma (69) indicating a possible functional variation for CD169^+^ TAMs between different tumor types.

Our findings also indicate that CD169^+^ Mo-M promote antibody and IL6 secretion from *in vitro* activated B cells, which hints in the direction that CD169^+^ macrophages actually may promote spontaneous germinal centre B cell formation locally (47). It is therefore likely that the association between CD169^+^ TAMs and TLLS, T cells and T_regs_ in breast tumors has a functional interrelation, where CD169^+^ TAMs could promote TLLS formations. The breast cancer patient cohort used in our study was from advanced breast cancer patients (23–26), where TLS and T_regs_ associated with a worse prognosis (22, 26), as did CD169^+^ TAMs (22). It is therefore interesting to note that the CD169^+^ Mo-M have functional similarities to lymph node resident CD169^+^ subcapsular sinus macrophages, with both B cell stimulatory and immunosuppressive potential (15–21, 35, 36, 49). This would indicate that the spatial colocalization with TLSs in primary breast tumors is not a coincidence and that CD169^+^ TAMs have unique functions in breast tumors. Alternatively, it is the B cells present in TLSs that could aid CD169^+^ TAMs differentiation as has been shown in lymph nodes (70).

In conclusion, we propose that CD169^+^ TAMs present in primary human breast cancer, are monocyte-derived macrophages generated in certain breast cancer microenvironments involving type I IFN signalling pathways. They possess immunosuppressive functions but simultaneously promote antibody and IL6 secretion by activated B cells. In advanced breast cancer patients, the CD169^+^ TAMs associate with TLSs containing T_regs_, with possible detrimental effects for these patients (22). The phenotypic and functional similarities between CD169^+^ Mo-M and lymph node resident CD169^+^ macrophages in cancer patients are intriguing and reflect a possible similar mode of action despite having opposite prognostic impact. The finding regarding opposite prognostic impact warrants further studies to understand whether lymph node resident CD169^+^ macrophages actually possess anti-tumorigenic features, or whether they rather disappear from late-stage lymph nodes containing metastasis. Their beneficial prognostic impact would then be related to CD169^+^ lymph node macrophages being present more often in lymph nodes of cancer patients with early-stage breast cancer, therefore linking them to a beneficial prognosis. More knowledge is therefore needed before we know whether CD169^+^ macrophages should be viewed as a therapeutic target.

## Data availability statement

Gene expression data were derived from the TCGA database in R2: Genomics Analysis and Visualization platform (www.hgserver1.amc.nl) and the public data set of Human breast tumor single cell RNA Seq data from the Michigan Portal for the Analysis of NGS Data (MiPanda) (https://mipanda.med.umich.edu/gene/Coexpression) (29).

## Ethics statement

The studies involving human participants were reviewed and approved by the regional Ethics Committees in Sweden; for the small breast cancer cohort from Lund Dnr 2010/477, and for the large clinical trial cohort Stockholm, Dnr KI 02-206 and KI 02-205 (22–26). A written informed consent was received from the included patients in the clinical trials presented in this study. The study was conducted in accordance with the Declaration of Helsinki. The patients/participants provided their written informed consent to participate in this study. The animal study was reviewed and approved by Djurförsöksetiska nämnden i Malmö/Lund Lund University, Sweden.

## Author contributions

The work reported in the paper has been performed by the authors. FG performed the majority of experiments with the help from RG, CR, MW and CM, while OB, AL and EK performed a substantial amount of the experiments. CA, and HV were responsible for the annotation of CD169, CD20, CD3 and CD56 IHC in the small TMA cohort. CH was responsible for collecting clinicopathological traits and outcome data for patients for the small TMA cohort. IH was responsible for collecting clinicopathological traits and outcome data for all patients for the large TMA cohort. MJ was responsible for professional guidance on performing and interpretations of IHC and annotations of TMAs. DB was responsible for performing the xenografts. OB and KL was responsible for managing the data analyses in SPSS. FG, OB, EK, CH, IH and KL were responsible for interpretation of final results. FG and KL was responsible for designing this study and drafting of the manuscript. All authors contributed to the article and approved the submitted version.
