# Supplementary material for: Breast cancer associated CD169+ macrophages possess broad immunosuppressive functions but enhance antibody secretion by activated B cells
Source: Front Immunol. 2023 Jun 19;14:1180209. doi: 10.3389/fimmu.2023.1180209 (PMC10315498; doi:10.3389/fimmu.2023.1180209)

Supplementary tables:

Supplementary Table 1. V Plex assay with cytokines secreted by M2 like macrophages and M2/type I IFN (CD169$^+$ Mo-M) macrophages measured.

Cytokine/ Chemokine	M2 (pg/ml)	M2/type I IFN (pg/ml)	P value*
CCL2	18058,81	26258,18	0,088
CCL3	4408,45	544,15	0,30
CCL4	14568,58	2313,65	0,074
CCL11	31,36	52,70	0,091
CCL13	8110,83	2588,21	0,13
CCL17	1266,14	1800,93	0,059
CCL20	16,71	16,55	0,95
CCL22	74285,06	26530,56	0,25
CCL26	28,38	41,09	0,52
CXCL10	48,96§	34823,64	**0,005**
IFNγ	0,78#	3,83#	0,36
IL1α	164,41	178,74	0,50
IL1β	193,23	256,41	0,25
IL2	-	-	-
IL5	-	-	-
IL6	1007,46	1332,13	0,079
IL7	4,83	5,34	0,80
IL8	5475,78	5507,61	0,55
IL10	429,43#	423,01#	0,36
IL12p70	0,37#	0,01#	0,36
IL-12/IL-23p40	82,81	78,31	0,64
IL13	4,56	6,23	0,25
IL15	9,19	10,43	**0,0002**
IL16	87,54	84,54	0,40
IL17A	10,50	8,81	0,48
IL21	15,54	13,20	0,43
IL22	-	-	-
IL23	-	-	-
IL27	81,80	87,73	0,15
IL31	-	-	-
TNFα	123,37	152,31	0,49
TNFβ	0,83	1,42#	0,73
VEGF	0,30#	-	-

- : Not detected

* P-value calculated by paired t-tests N=6

Undetectable in 5 out of 6 samples

§ Value below lowest standard but measurable

Supplementary Table 2. Flow cytometry antibodies

Antibody	Fluorochrome	Clone
CD169	PE	7-239
CD169	Alexa fluor 647	7-239
CD14	PECy7	M5E2
CD14	FITC	M5E2
HLA-DR	FITC	L243
IL15Ra	PE	JM7A4
IL15	APC	34559
CD1a	PE	HI149
CD206	APC	19.2
PDL1	APC	MIH1
CD163	PE	GHI/61
CD80	PE	L307.4

Supplementary Table 3. Primer sequences for RT-qPCR

Gene	Sequence
SIGLEC1	F: 5'-GGCTGTTACGATGGTTTATGATGT-3'<br>R: 5'-AATCAAAGGCATCATTTTAGGGATA-3'
IFNA	F: 5'-GACTCCATCTTGGCTGTGA-3'<br>R: 5'-TGATTTCTGCTCTGACAACCT-3'
IFNB	F: 5'-TTGACATCCCTGAGGAGATTAAGC-3'<br>R: 5'-TTGACATCCCTGAGGAGATTAAGC-3'
PTGES2	F: 5'-AGACGGACCACCTCATTCTC-3'<br>R: 5'-GCCTAAGGATGGCAAAGACC-3'
IDO1	F: 5'-CAAAGGTCATGGAGATGTCC-3'<br>R: 5'-CCACCAATAGAGAGACCAGG-3'
HLA-G	F: 5'-TGGAGCAGGAGGGGCCGGAG-3'<br>R: 5'-CCGCGCAGGGTCTGCAGGTT-3'
ARG1	F: 5'-GGCAATTGGAAGCATCTCTGGC-3'<br>R: 5'-AGTGTTCCCCAGGGTCC-3'
iNOS	F: 5'-GAGATCAACATTGCTGTGATCCATAG-3'<br>R: 5'-CACGGGACCGGTATTCATTC-3'
SDHA	F: 5'-TGGGAACAAGAGGGCATCTG-3'<br>R: 5'-CCACCACTGCATCAAATTCATG-3'
GAPDH	F: 5'- TGCACCACCAACTGCTTAGC-3'<br>R: 5'- GGCATGGACTGTGGTCATGAG-3'
CXCL10	F: 5'-GGTGAGAAGAGATGTCTGAATCC-3'<br>R: 5'-GTCCATCCTTGGAAGCACTGCA-3'
VISTA	F: 5'-AGATGCACCATCCAACTGTGTGG-3'<br>R: 5'-AGGCAGAGGATTCCTACGATGC-3'
Ox40L<br>*CD252*	F: 5'-CCTACATCTGCCTGCACTTCTC-3'<br>R: 5'-TGATGACTGAGTTGTTCTGCACC-3'
YWHAZ	F: 5'-ACTTTTGGTACATTGTGGCTTCAA-3'<br>R: 5'-CCGCCAGGACAAACCAGTAT-3'
STING	F: 5'-CCTGAGTCTCAGAACAACTGCC-3'<br>R: 5'-GGTCTTCAAGCTGCCCACAGTA-3'
IL10	F: 5'-CCCTGGGTGAGAAGCTGAAG-3'<br>R: 5'-CACTGCCTTGCTCTTATTTTCACA-3'

Supplementary Table 4. Cross-correlation CD169$^+$ tumor associated macrophages (CD169 PT) and tertiary lymphoid like structures (TLLS PT) or NK cells in small test breast cancer cohort.

		**CD169 PT**				**Total**	***P***
		**0**	**1**	**2**	**3**		
**TLLS PT**	***0***	1	12	7	0	20	
	1	0	2	1	0	3	
	2	0	0	0	1	1	
Total		1	14	8	1	24[a]	*P*=0.048
**NK**	***0***	1	7	4	0	12	
	1	0	7	3	1	11	
Total		1	14	7	1	23[a]	*P*=0.449

[a] Pearson Chi-Square, Linear by Linear association

Supplementary Table 5. Cross-correlations for spatial association between CD169^{+} tumor associated macrophages (CD169 PT) and T cells (CD3) in primary breast tumors using the large breast cancer cohort (Kimbung et al., 2016).

		**CD169 PT**		**Total**	***P***
		**Neg**	**Pos**		
**CD3 PT**	***low (0-1)***	85	22	107	
	high (2-3)	43	25	68	
Total		128	47	175[a]	*P= 0.018*

[a] Pearson Chi-Square, Linear by Linear association
*Fisher's exact test was used when fewer observations than 20 were seen in at least 1 category

Supplementary Figure 1.

Phenotype of primary human monocyte-derived macrophages. (A) Tumor xenografts in NSG mice. Primary human monocytes were co-transplanted with MDA-MB-231 breast cancer cell lines in NSG mice for 21 d. Controls were transplanted with MDA-MB-231 cells alone. Upregulation of CD169 (red) was only seen in the SUM159 + monocyte xenografts (Fig. 2A-B) but not MDA-MB-231 + monocytes (this Figure S1A), while PDL1 (brown) was seen in all xenografts SUM159 (Fig. 2A-B), SUM159 + monocyte (Fig. 2A-B), MDA-MB-231 (this Figure S1A), MDA-MB-231 + monocytes (this Figure S1A) xenografts. (B) Relative mRNA levels of *IFNA*, *IFNB* and *IFNG* in primary human monocytes (Mo) and Mo-M as measured by RT-qPCR, N = 3, one-way ANOVA multiple comparison Dunnett's test. Note, the donors for Mo are not the same as for macrophages (M1, M2, M2/IFN) in this experiment. (C) *SIGLEC1* association to *IFNA4* (left) and *INFB1* (right) mRNA expression in primary tumors of breast cancer patients using the TCGA data base in R2 (r2.amc.nl) in R2: Genomics Analysis and Visualization platform (www.hgserver1.amc.nl). (D) PDL1 surface expression of primary human Mo-M (left), N = 4, Paired t-test. PDL1 expression on $CD169^{+}$ cells (middle) and $CD169^{-}$ cells (right), N = 4. (E) Ratio of median MFI of CD14 surface expression, N = 3, one-way ANOVA multiple comparison Dunnett's test. (F) Flow cytometry dot plots for HLA-DR and CD14 and MFI histograms for CD206 and CD1a to show gating strategy.

Figure S1

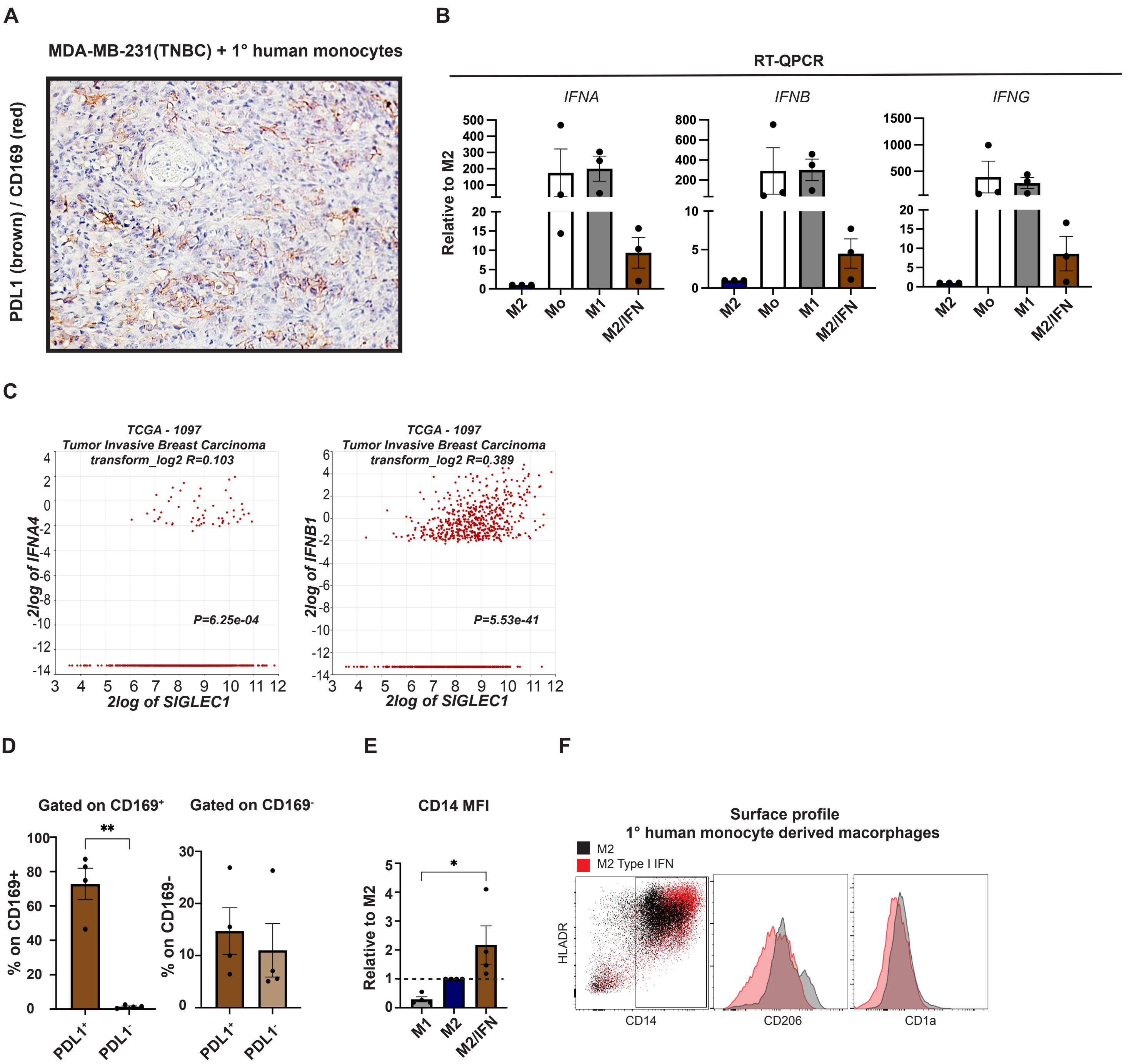

A
MDA-MB-231(TNBC) + 1° human monocytes
PDL1 (brown) / CD169 (red)
B
RT-QPCR
IFNA
IFNB
IFNG
Relative to M2
M2
Mo
M1
M2/IFN
C
TCGA - 1097
Tumor Invasive Breast Carcinoma
transform_log2 R=0.103
P=6.25e-04
2log of IFNA4
2log of SIGLEC1
TCGA - 1097
Tumor Invasive Breast Carcinoma
transform_log2 R=0.389
P=5.53e-41
2log of IFNB1
2log of SIGLEC1
D
Gated on CD169+
Gated on CD169-
% on CD169+
% on CD169-
PDL1+
PDL1-
**
E
CD14 MFI
Relative to M2
*
M1
M2
M2/IFN
F
Surface profile
1° human monocyte derived macorphages
M2
M2 Type I IFN
HLADR
CD14
CD206
CD1a

Supplementary Figure 2

Functional phenotype of CD169^{+} Mo-M. (A) Cytokine secretion of IL6 as measured by CBA, ratio of concentration with M2 as control, N = 6, Ratio paired t-test. (B) TNFα cytokine secretion measured with CBA, N = 3. Student's t-test. (C) Allogeneic co-culture of primary human NK cells, primary human monocyte-derived macrophages and MDA-MB-231 breast cancer cell line with inhibitors for HLA-G (10 μg/ml) and PDL1 (Atezolizumab, 10 μg/ml), N = 6. (D) T_{reg} cell differentiation cultures of primary human monocyte-derived macrophages and primary human CD4^{+} T cells as measured by RT-QPCR of *FOXP3* expression N=9 and N=3 ctrl. One-way ANOVA multiple comparison Dunnett's test. (E) IL10 secretion measured in B cell differentiation cultures of primary human monocyte-derived macrophages and primary human B cells as measured by CBA, N=6. Ratio paired t-test. For all figures: error bars indicate SEM. * $p < 0.05$, ** $p < 0.01$, *** $p < 0.001$, **** $p < 0.0001$.

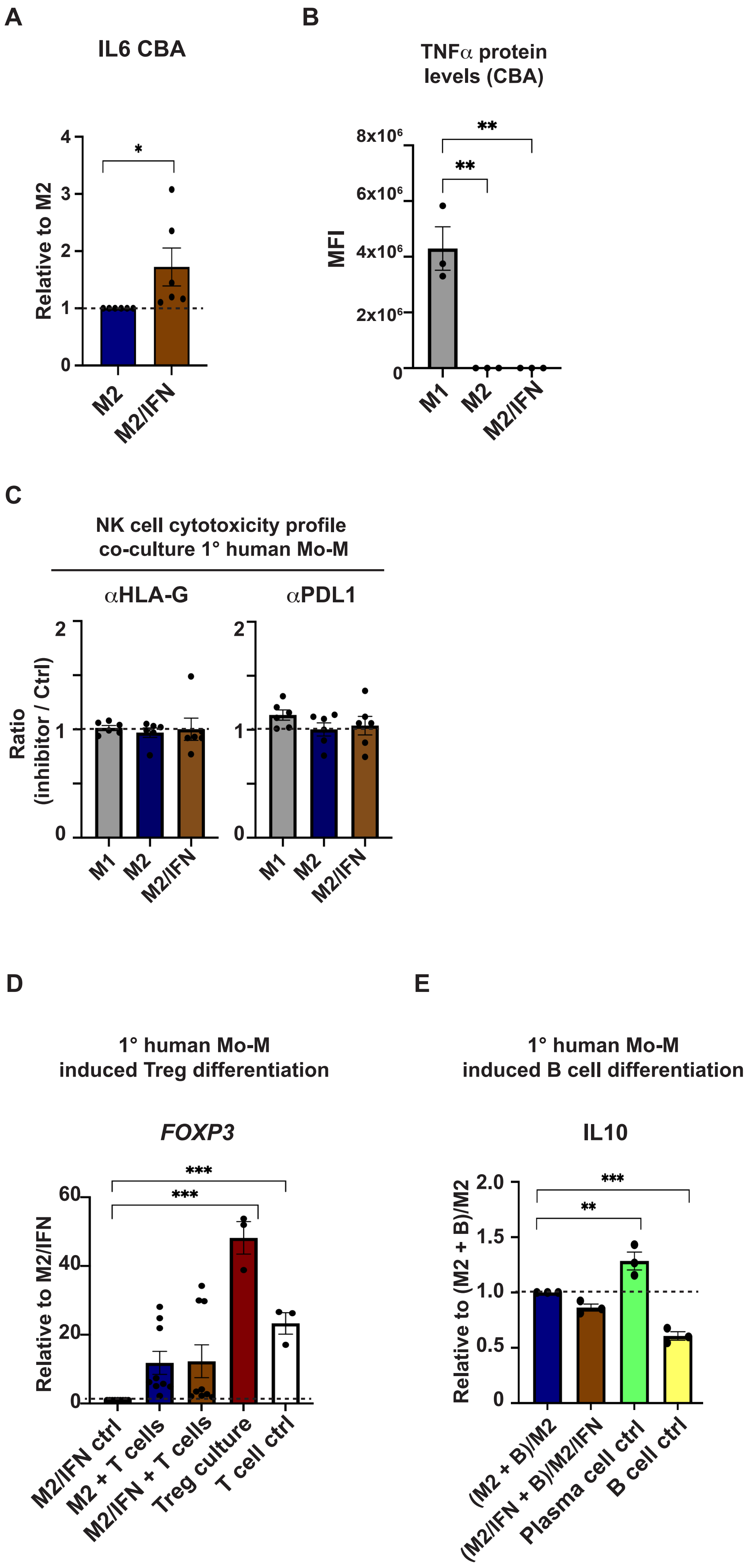
A
IL6 CBA
Relative to M2
*
M2
M2/IFN
B
TNFα protein levels (CBA)
MFI
8x10^6
6x10^6
4x10^6
2x10^6
**
**
M1
M2
M2/IFN
C
NK cell cytotoxicity profile co-culture 1° human Mo-M
αHLA-G
αPDL1
Ratio (inhibitor / Ctrl)
M1
M2
M2/IFN
D
1° human Mo-M induced Treg differentiation
FOXP3
Relative to M2/IFN
***
***
M2/IFN ctrl
M2 + T cells
M2/IFN + T cells
Treg culture
T cell ctrl
E
1° human Mo-M induced B cell differentiation
IL10
Relative to (M2 + B)/M2
***
**
(M2 + B)/M2
(M2/IFN + B)/M2/IFN
Plasma cell ctrl
B cell ctrl

Supplementary Data File 1

The flow cytometry gating strategies are shown for (A) CD14 gated CD169+PDL1+ cells (B) CD169 MFI on CD14 gated M1, M2 and M2/IFN Mo-Ms (C) HLADR and CD14 MFI (D) CD14 gated CD163+CD169+ Mo-M (E) live gated CD80 MFI.

Representative gateing strategies for flow cytometric analyses

A

Gateing strategy for CD169+PDL1+

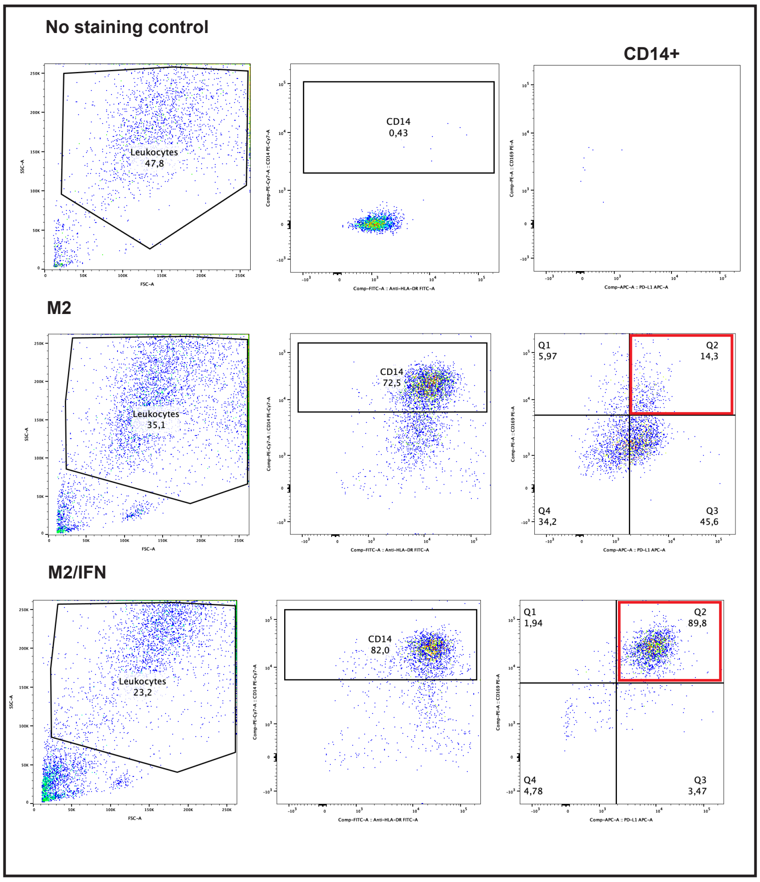

B

Gated on CD14

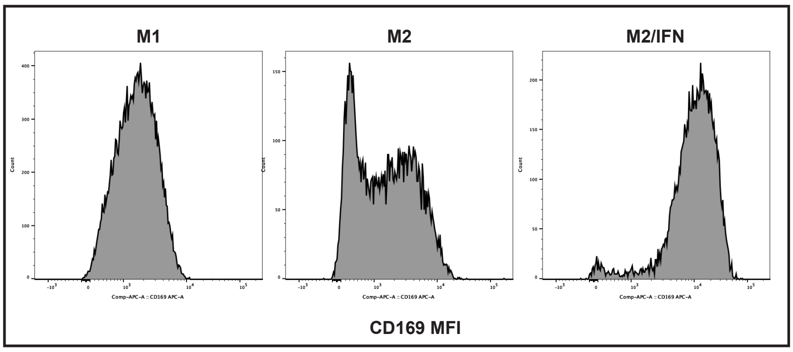

C

Gate for CD14 and HLADR MFI

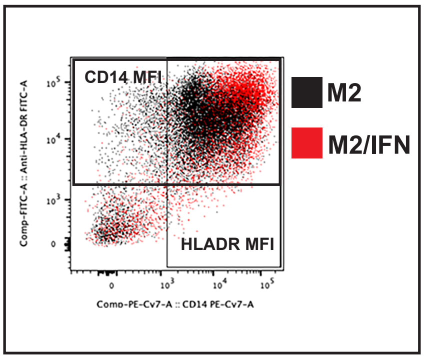

D

Gateing strategy for CD169+CD163+

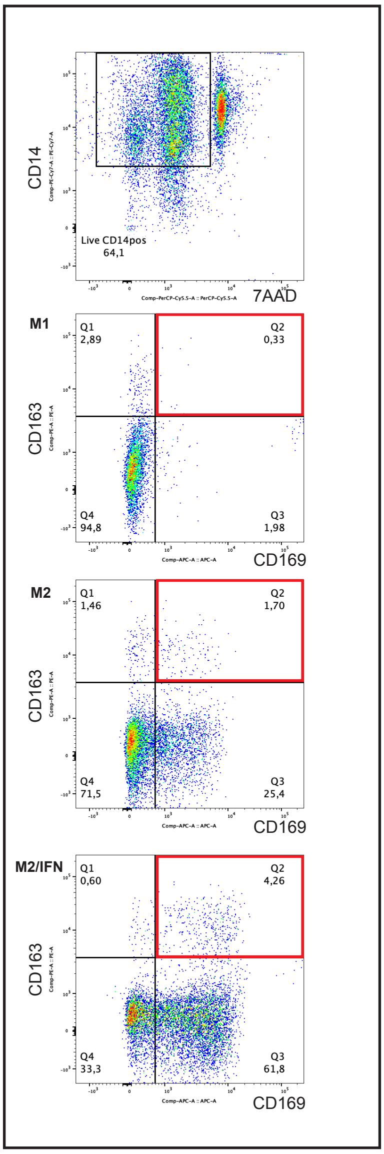

E

Gateing strategy for CD80 MFI

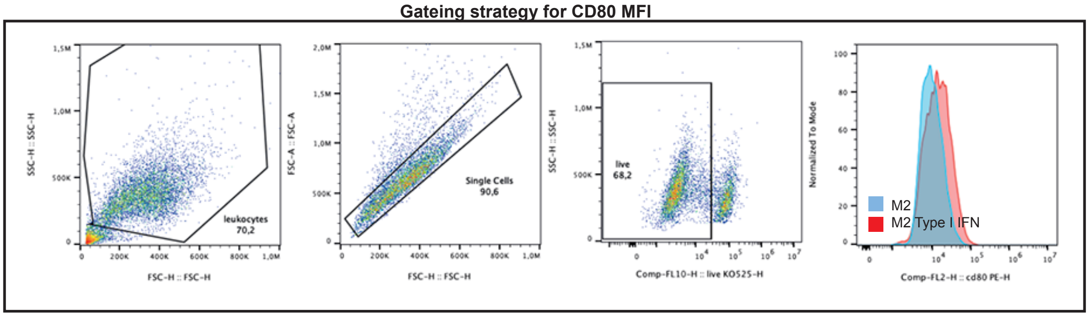

Supplement: Supplementary file 1 [file DataSheet_1.pdf]
